# Diels–Alder reactions of myrcene using intensified continuous-flow reactors

**DOI:** 10.3762/bjoc.13.15

**Published:** 2017-01-19

**Authors:** Christian H Hornung, Miguel Á Álvarez-Diéguez, Thomas M Kohl, John Tsanaktsidis

**Affiliations:** 1 CSIRO Manufacturing, Bag 10, Clayton South, Victoria 3169, Australia

**Keywords:** continuous processing, flow chemistry, renewable feedstock, surfactant

## Abstract

This work describes the Diels–Alder reaction of the naturally occurring substituted butadiene, myrcene, with a range of different naturally occurring and synthetic dienophiles. The synthesis of the Diels–Alder adduct from myrcene and acrylic acid, containing surfactant properties, was scaled-up in a plate-type continuous-flow reactor with a volume of 105 mL to a throughput of 2.79 kg of the final product per day. This continuous-flow approach provides a facile alternative scale-up route to conventional batch processing, and it helps to intensify the synthesis protocol by applying higher reaction temperatures and shorter reaction times.

## Introduction

**Scheme 1:**
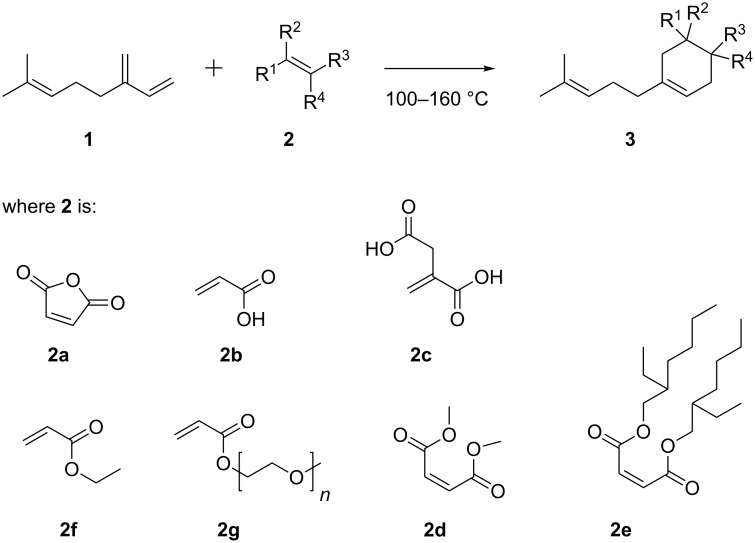
Diels–Alder reaction of myrcene (**1**), with various dienophiles **2**.

Over the past years, great attention has been devoted to finding alternative, renewable feedstocks to fossil oil for the production of fuel and industrial chemicals. Especially, high value added products from fine chemicals, specialty chemicals or the pharmaceuticals sector allow for a ‘drop-in’ replacement of existing, fossil resources based synthesis routes with economic alternatives based on renewable sources. Besides chemical platforms based on sugar, lignin or fatty acid containing feedstocks, terpenes present another plant derived feedstock which is of great interest for a variety of industrial applications, first and foremost in the fragrance and flavor industries, but also in the pharmaceutical and chemical industries [1–3]. Myrcene is a naturally occurring, acyclic monoterpene which is used industrially for the manufacture of flavoring substances and fragrances; in research it is used as a model compound for a series of different reactions and in the synthesis of complex natural products, including several pheromones [3]. Myrcene is a colorless oil and exists as two isomers, the synthetic α-myrcene, containing an isopropenyl group, and the naturally occurring β-myrcene (which will be referred to in the following only as “myrcene” (**1**), see Scheme 1, vide infra). It can be found in significant quantities (up to 39%) in the essential oils of several plants, such as wild thyme [4], ylang-ylang [5], bay leaf [6], juniper berries [7], lemongrass [8], or parsley [9], and in smaller percentages (<5%) in hops [3], celery [3], dill [9], rosemary [3], tarragon [10] and nutmeg [3] to name but a few. A review by Behr and Johnen [3] describes the manufacture of myrcene from other terpenes, as well as several synthetic routes based on this versatile and reactive starting material to form alcohols, esters, amines, chlorides, dimers, polymers and even complex natural products, amongst others. At present myrcene (**1**) is manufactured industrially from turpentine; the distillate of pine resin [3]. One of the main components of turpentine is β-pinene, from which myrcene can be synthesized upon thermal isomerization at temperatures between 400 and 600 °C. This was first described by Goldblatt and Palkin in 1947 [11]. Myrcene is a very versatile molecule that can act as the starting material for several valuable compounds. The industrial production of a series of top-selling flavors and fragrances are based on myrcene, such as geraniol, nerol, linalool, menthol, citral, citronellol or citronellal [3]. The terminal diene moiety present in myrcene allows for a reaction with a suitable dienophile following the Diels–Alder reaction mechanism. Dahill et al. describe the synthesis of the Diels–Alder adduct of myrcene and acrylonitrile for the use as an odorant in the perfume industry [12]. A series of Diels–Alder reactions of myrcene (**1**) and another sesquiterpene, farnesene, with various dienophiles have been reported by Tabor et al. [13] for the use as solvents and surfactants.

The emergence of compact continuous-flow reactors has begun to transform the way chemical synthesis is conducted in research laboratories and small manufacturing over the past few years [14–21]. In several applications, where reaction times are short and heat management is important, intensified continuous processes inside tubular or plate-type flow reactors can successfully replace batch methodologies classically carried out in stirred glass vessels. We have demonstrated the benefits of this superior heat management in previous work looking at exothermic radical polymerizations in continuous flow [22–23]. Over the past years, Diels–Alder reactions of isoprene using laboratory-scale flow reactors were studied by different research groups [24–25]. A continuous-flow reactor can offer a range of benefits over batch processing, with the enhanced heat and mass transfer arguable being one of the most important. In many cases increased control over the process and improvements in product quality are the result. Herein, we describe the synthesis of several Diels–Alder adducts made from myrcene (**1**) and a series of dienophiles, which contain carboxylic acids, esters or acid anhydrides. In particular, the reaction of myrcene (**1**) with acrylic acid (**2b**) was investigated in detail, through batch and continuous-flow methods. The intensified flow process presents a more compact and efficient alternative to classic batch manufacture for the production of Diels–Alder adduct surfactants from myrcene.

## Results and Discussion

The solution-phase Diels–Alder reactions presented herein follow the general reaction pathway shown in Scheme 1. The conjugated diene myrcene (**1**) was reacted with a series of dienophiles **2** to form the Diels–Alder adducts **3**.

Before investigating this reaction for continuous-flow processing, we first undertook a series of batch experiments to explore the reactivity of the different dienophiles shown in Scheme 1. These experiments were carried out on a batch microwave-reactor system (see experimental section) at temperatures between 100 and 140 °C, and the results are presented in Table 1.

**Table 1:** Reagents, reaction conditions and results for small scale batch reaction of myrcene with various dienophiles.

entry	dienophile	solvent^a^	*T* [°C]	reaction time	conversion [%]^b^

1.1	**2a**	THF	100	5 min	90
1.2	**2b**	toluene	140	1 h	98
1.3	**2c**	iPrOH	140	10 h	70
1.4	**2d**	neat	140	10 h	97
1.5	**2e**	neat	140	10 h	93
1.6	**2f**	neat	120	5 h	96
1.7	**2g**	neat	140	10 h	48

^a^Entries 1.1 to 1.3 were reacted with an initial myrcene concentration, *c*_MYR,0_, of 2.8 mol/L; all entries were reacted with a myrcene to dienophile ratio, *R*, of 0.9; ^b^conversions were calculated based on NMR.

**Figure 1:**
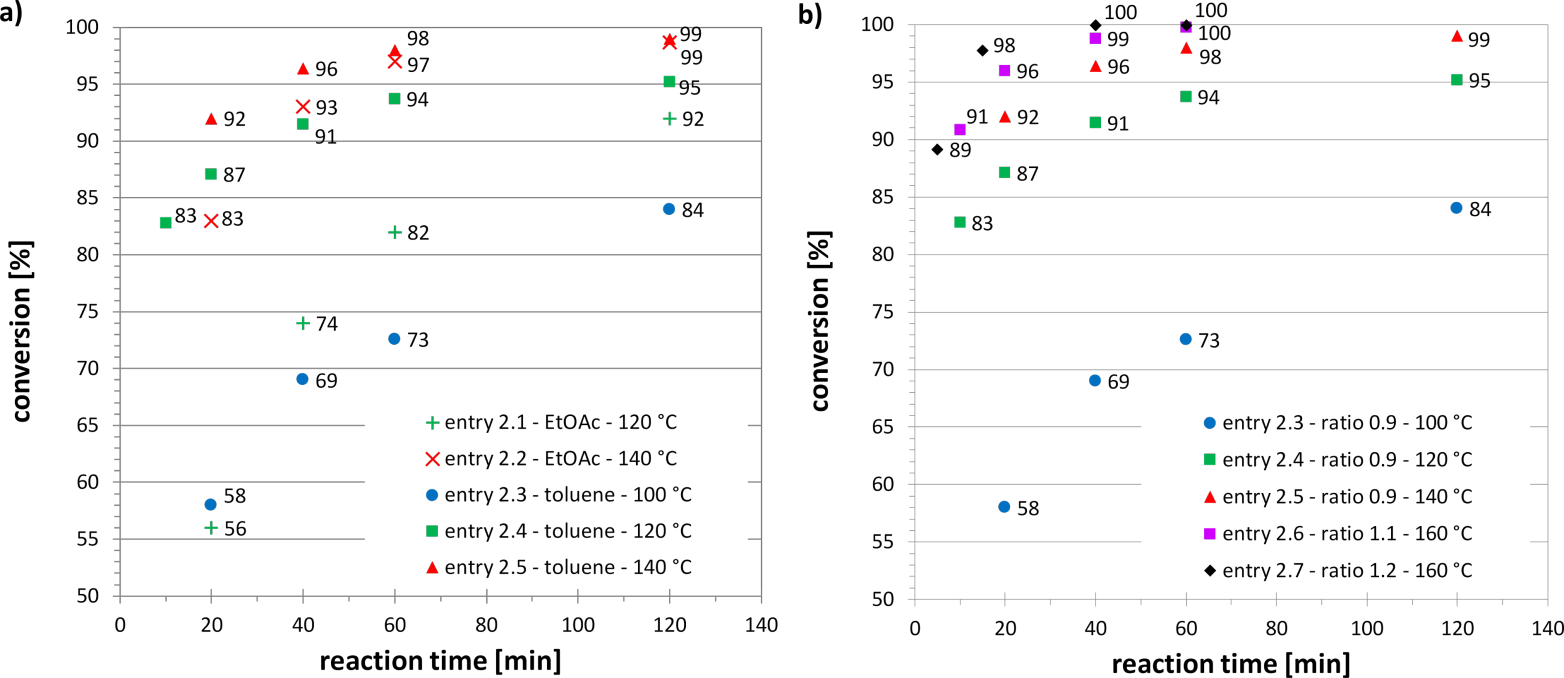
Kinetic studies of the Diels–Alder reaction between myrcene (**1**) and acrylic acid (**2b**); a) for different solvents and temperatures, ratio of myrcene to acrylic acid: 0.9; b) for different starting material ratios and temperatures, solvent: toluene.

Maleic anhydride (**2a**) proved to be the most reactive of the dienophiles used in this study with reaction completion occurring after a few minutes at 100 °C. Other activated dienophiles such as acrylic acid (**2b**) and ethyl acrylate (**2f**) reached high conversions in excess of 90% after 1 to 5 h and the maleates **2d** and **2e** required up to 10 h reaction time at 140 °C to reach near-completion. The slowest reactions were observed using itaconic acid (**2c**) and the PEG containing acrylate **2g**. Acrylic acid (**2b**) was selected for further study given our interest in products with surfactant properties, and the preferable reaction kinetics of the acrylic acid–myrcene system. Table 2 presents a set of experiments using this system, at different process conditions and in different solvents; samples were analyzed over time in order to establish kinetic profiles of these reactions. Figure 1 shows the kinetic profiles of the reactions presented in Table 2.

**Table 2:** Solvents, reaction conditions, conversions and reaction rate constants, *k*, for small scale batch reactions of myrcene (**1**) with acrylic acid (**2b**); for further details on derivation of *k* values see Supporting Information File 1.

entry	solvent	*c*_MYR,0_ [mol L^−1^]^a^	*R* [–]^b^	*T* [°C]	reaction time [h]	conversion [%]^c^	*k* × 10^3^ [L mol^−1^ s^−1^]^d^

2.1	EtOAc	2.8	0.9	120	2	92	0.53
2.2	EtOAc	2.8	0.9	140	2	99	3.44
2.3	toluene	2.8	0.9	100	2	84	0.27
2.4	toluene	2.8	0.9	120	2	95	1.14
2.5	toluene	2.8	0.9	140	2	99	4.75
2.6	toluene	2.9	1.1	160	1	~100	27.05
2.7	toluene	2.9	1.2	160	1	~100	–

^a^Initial myrcene concentration; ^b^ratio of myrcene to acrylic acid; ^c^conversions were calculated based on NMR; ^d^*k* was derived from kinetic studies plotted in Figure 1 for entries 2.1 to 2.6, as in these experiments *R* was close to 1 (between 0.9 and 1.1).

All reactions followed an expected trend, asymptotically approaching full conversion with increasing reaction time. While both EtOAc and toluene produced similarly fast kinetic data with conversions around 95% after 40 to 60 min toluene was preferred due to its higher boiling point. Figure 1b shows the influence of temperature and the ratio of starting materials. These experiments also showed trends as were expected. Values for the reaction rate constant, *k*, calculated from these experiments, are presented in Table 2 and are within expected limits when compared to literature values. More details on the derivation of the *k* values and the literature references can be found in Supporting Information File 1. After the Diels–Alder reaction was optimized in batch on a small scale (typically 2 mL reaction volume) the process was scaled-up first on a Vapourtec R2/R4 tubular flow reactor to a reaction volume of typically 20 mL and then on a Chemtrix Plantrix^®^ MR260 plate flow reactor to a reaction volume of typically 200 mL (see also experimental section). The results from these continuous-flow experiments are shown in Table 3.

**Table 3:** Solvents, reaction conditions and results for the continuous-flow reaction of myrcene (**1**) with acrylic acid (**2b**) in a tubular flow reactor (reactor volume: 20 mL) and a plate flow reactor (reactor volume: 105 mL); all entries were reacted with a myrcene to dienophile ratio, *R*, of 0.9, and *c*_MYR,0_ of 2.8 mol/L.

entry	reactor	solvent	*R* [–]	*T* [°C]	residence time [min]	conversion [%]^a^

3.1	tubular	EtOAc	0.9	140	20	75
3.2	tubular	EtOAc	0.9	140	30	95
3.3	tubular	EtOAc	0.9	140	40	99
3.4	tubular	toluene	0.9	120	40	93
3.5	tubular	toluene	0.9	140	40	99
3.6	plate	toluene	0.9	112	40	85
3.7	plate	toluene	0.9	130	40	93
3.8	plate	toluene	1.1	140	40	~100
3.9	plate	toluene	1.1	160	30	99

^a^Conversions were calculated based on NMR.

**Figure 2:**
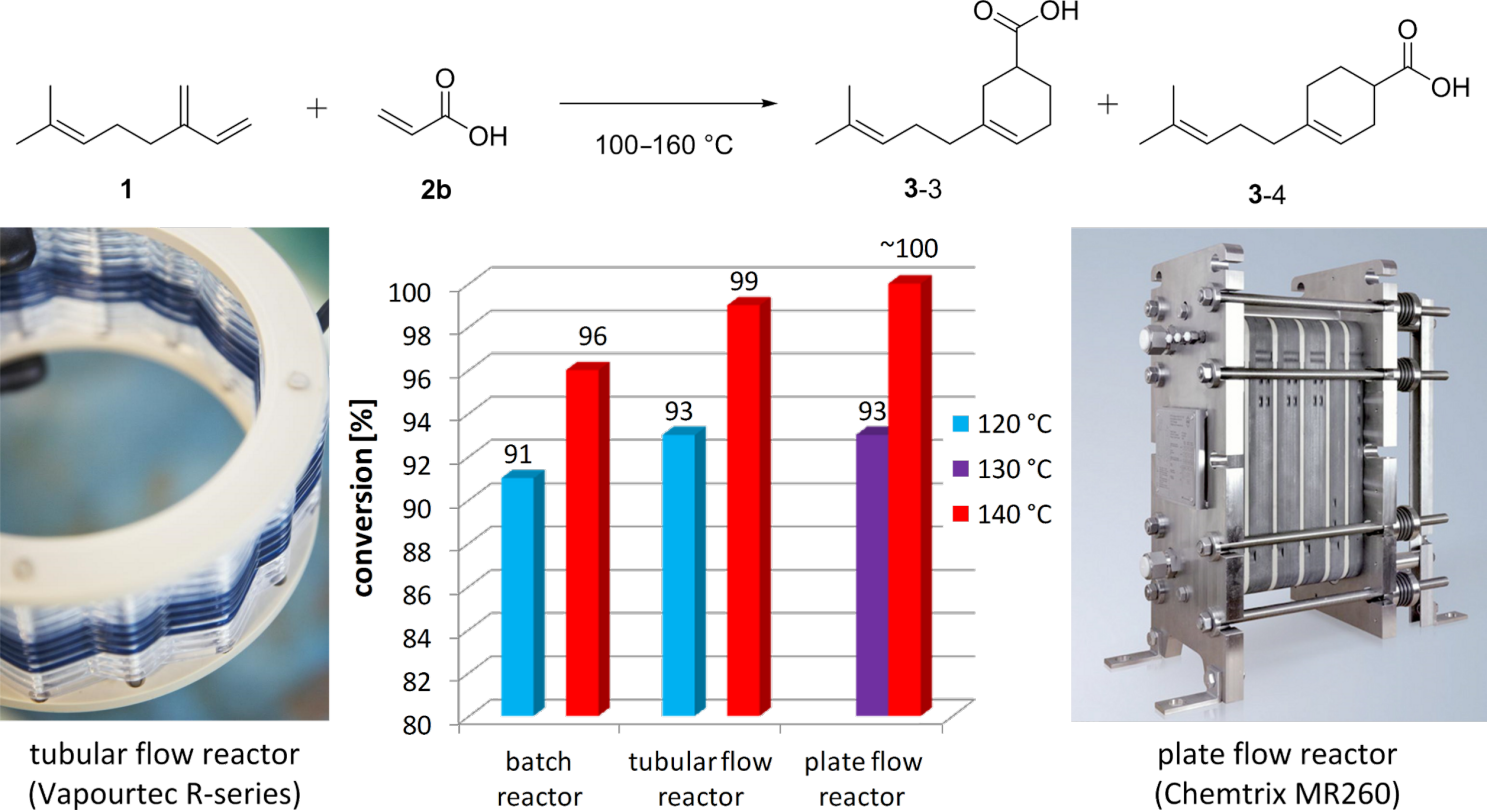
Comparison of conversions in three different reactors for the Diels–Alder reaction of myrcene (**1**) with acrylic acid (**2b**) in toluene; the reaction forms two isomers, **3**-3 and **3**-4; reaction temperature for these experiments: 120, 130 or 140 °C, reaction time: 40 min; photographic images of a tubular reactor coil of the Vapourtec R2/R4 flow reactor [26] and of the plate reactor module of the Chemtrix Plantrix^®^ MR260 [27].

The 10-times scale-up in the tubular flow reactor and the 100 times scale-up in the plate flow reactor resulted in similar, if not slightly higher conversions than the batch experiments (see Figure 2). The two continuous reactors produced high-quality material at steady state conditions. The reaction profile in the plate flow reactor was quantified by taking samples at the outlet of the reactor over the entire duration of one experiment. These profiles are very uniform with steep fronts and tails and a flat steady state region, suggesting that the residence time distribution inside the reactor is narrow and close to plug flow. One of these profiles is shown in Figure S4 (Supporting Information File 1). The fastest conditions investigated herein were 30 min in the plate reactor at 160 °C giving 99% conversion of **2b** and a yield of 94% of a semi-crystalline product (Table 3, entry 3.9). As part of the scale-up investigations, we also performed the Diels–Alder reaction of myrcene (**1**) and **2b** in a 6 mm i.d. stainless steel tubular flow reactor with a reaction volume of 108 mL. A few minutes after start of the reaction, however, we observed a pressure increase in the reactor which was caused by fouling occurring in the reactor entrance section and ultimately led to complete blockage of the tube at this point. This is believed to be caused by a side reaction of **2b** and myrcene (**1**) forming polymeric material, which built up on the metal walls of the reactor, ultimately leading to the complete blockage. The mechanism and circumstances of this side-reaction are unknown; it only occurred in the stainless steel reactor and not in the PFA tubing of the Vapourtec R-series flow reactor or the silicon carbide module of the plate flow reactor. Hence, it was postulated that a metal catalyzed polymerisation on the stainless steel reactor tubes might have occurred, however, this could not be confirmed. Further details on these observations can be found in Supporting Information File 1.

Using ^13^C NMR an approximate ratio of the two isomers, **3**-3 and **3**-4 (see Figure 2), was calculated for the continuous-flow reactions performed between 140 and 160 °C (see Table 3). The amount of Diels–Alder adduct with the carboxylic acid located in the 3-substituted position, **3**-3, was always larger than the 4-substitituted adduct, **3**-4, with an average **3**-3/**3**-4 ratio of 7:3 (3-substituited adduct was between 68 and 71%).

For Table 3, entry 3.9, the yield of the semi-crystalline product after solvent removal was 94%. The production capacity (PC) and the space time yield (S.T.Y.) can be calculated based on the amount of isolated product, *m*_P_, using Equations 1 and 2.

[1]
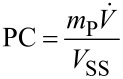


[2]
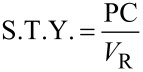


Here, 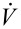
is the total volumetric flow rate through the reactor, *V_SS_* the combined volume of both stock solutions and *V_R_* the volume of the flow reactor. Running the plate reactor at 160 °C (Table 3, entry 3.9), we managed to achieve a production capacity of 116.3 g/h, which equates to an S.T.Y. of 1.11 kg L^−1^ h^−1^. Parallel to the scale-up in the plate flow reactor, we also scaled up the process in batch to a 6 L scale using a jacketed stirred tank reactor. Here, the reaction was run for ~10 h at 100 °C in order to reach completion, compared to only 30 min at 160 °C in continuous flow.

Preliminary experiments were carried out looking at the surfactant properties of the Diels–Alder adduct of myrcene (**1**) and **2b**. The results were promising and showed that the product was able to stabilize emulsions for several hours compared to several seconds or minutes in the control experiments without the Diels–Alder adduct. Further details on these surfactant tests are presented in Supporting Information File 1.

## Conclusion

We have investigated the Diels–Alder reaction of myrcene (**1**) with a range of different dienophiles at temperatures between 100 and 160 °C. The Diels–Alder reaction of myrcene (**1**) with acrylic acid (**2b**), yielding a carboxylic acid containing surfactant, was scaled-up in a plate-type continuous-flow reactor and a batch stirred tank. The use of continuous-flow processing allows for an efficient synthesis of large quantities of the Diels–Alder adduct and we managed to scale-up the reaction of myrcene (**1**) with acrylic acid (**2b**) inside the 105 mL flow reactor to a throughput of 2.79 kg of the final product per day. The small dimensions of the fluidic channels inside the tubular and the plate-type flow reactors ensured that heat and mass transfer were efficient and fast, and that the reaction could be operated under ‘quasi isothermal’ conditions (i.e., with negligible deviations from the set temperature in the entire bulk reaction volume of the reactor). This resulted in a much more uniform reaction profile than in batch stirred tanks, allowing for a much shorter reaction time than classically applied in batch operations.

## Experimental

### Materials and analysis

The reactants myrcene (**1**, 90% purity), maleic anhydride (**2a**), acrylic acid (**2b**), itaconic acid (**2c**), dimethyl maleate (**2d**), ethyl acrylate (**2f**) and poly(ethylene glycol) methyl ether acrylate (PEGA, **2g**) were obtained from Sigma-Aldrich; bis(2-ethylhexyl) maleate was provided by TriTech Lubricants. The solvents tetrahydrofuran (THF), ethyl acetate (EtOAc), toluene, dichloromethane (DCM) and isopropanol (iPrOH) were obtained from Merck KGaA. All reagents and solvents were used without further purification.

Reaction conversions were calculated from ^1^H NMR spectra, which were recorded on a Bruker AC-400 spectrometer in deuterated chloroform (from Cambridge Isotope Laboratories Inc.). Conversion calculations were based on clearly identifiable and non-convoluted peaks of remaining starting material and generated product. The residual solvent peak at δ = 7.26 ppm was used as an internal reference. Product compositions were analyzed by GC–FID and GC–MS; details for both can be found in Supporting Information File 1. The GC–FID results were also used to confirm NMR conversions and to calculate GC-based yields.

### Batch Diels–Alder reaction

The following procedure is typical for the preparation of the Diels–Alder adduct of myrcene (**1**) and a series of different dienophiles. A reactant solution of myrcene (**1**, 811 mg of myrcene stock solution with a 90% purity, 5.36 mmol of myrcene), **2b** (429 mg, 5.95 mmol), in EtOAc (0.49 mL), was premixed and filled into a sealed microwave vial. The reaction was conducted in a laboratory microwave reactor (Biotage Initiator) at 140 °C with a reaction time of 2 h. A transparent, faintly yellow solution was obtained after reaction, from which the conversion was determined by ^1^H NMR. The solvent was evaporated under reduced pressure to yield a yellow semi-crystalline paste. Detailed reaction conditions and reagent compositions for each batch experiment can be found in Table 1 and Table 2. For kinetic studies, small samples of the reaction mixture for ^1^H NMR were withdrawn through the septum of the microwave reactor glass vial using a syringe. For this the microwave reaction was stopped at various points in time over the course of the reaction, namely at 20, 40, 60 and 120 min.

### Continuous-flow Diels–Alder reaction using a Vapourtec R2/R4 flow reactor

The following procedure is typical for the preparation of the Diels–Alder adduct of myrcene (**1**) and acrylic acid (**2b**) in a tubular flow reactor. Two reactant solutions were prepared, one containing myrcene (16.22 g of myrcene stock solution with a 90% purity, 107.16 mmol of myrcene) in EtOAc (1.98 mL), and the other containing **2b** (8.58 g, 119.06 mmol), in EtOAc (7.75 mL). The two solutions were continuously mixed in a T-piece and then fed into a Vapourtec R2/R4 flow reactor set-up [26], consisting of two 1.0 mm i.d. perfluoroalkoxy alkane (PFA) reactor coil modules in series (10 mL each – total reactor volume: 20 mL). The pump flow rate of the myrcene solution was set to 0.3 mL∙min^−1^, the pump flow rate of the acrylic acid solution was set to 0.2 mL∙min^−1^. This resulted in a total flow rate of 0.5 mL∙min^−1^ and a mean hydraulic residence time of 40 min inside the two PFA reactor coils (the mean hydraulic residence time is defined as ‘flow rate/reactor volume’). The reaction was conducted at 140 °C. The product, a transparent, faintly yellow solution, was collected at the reactor outlet, after passing through a 75 psi back-pressure regulator. From this solution, the reaction conversion was determined by ^1^H NMR. Afterwards, the solvent was evaporated under reduced pressure to yield a yellow semi-crystalline paste. Detailed reaction conditions and reagent compositions for each experiment in the tubular flow reactor can be found in Table 3.

### Continuous-flow Diels–Alder reaction using a Chemtrix MR260 flow reactor

The following procedure is typical for the preparation of the Diels–Alder adduct of myrcene (**1**) and acrylic acid (**2b**) in a silicon carbide plate-type flow reactor. Two reactant solutions were prepared, one containing myrcene (208.2 g of myrcene stock solution with a 90% purity, 1.375 mol of myrcene) in toluene (21.2 mL), and the other containing **2b** (90.1 g, 1.250 mol), in toluene (80.1 mL). The two feed solutions were pumped using two Teledyne Isco D-series dual syringe pumps (100 DX, with Hastelloy™ syringes) and were continuously mixed in a T-piece. After mixing, the combined starting material solution was fed into a Chemtrix Plantrix^®^ MR260 [27] plate-type flow reactor. This plate flow reactor configuration consisted of a series of 3M™ silicon carbide microstructured plates (see also Figures S2 and S3 in Supporting Information File 1), which was thermally regulated by a Lauda Integral XT 150 heater/chiller unit. The total reactor volume was 105 mL. An SSI Prep 100 dual piston pump with PEEK pump heads was used to flush the reactor before and after the reaction with toluene. The pump flow rate of the myrcene solution was set to 2.21 mL∙min^−1^, the pump flow rate of the acrylic acid solution was set to 1.30 mL∙min^−1^. This resulted in a total flow rate of 3.51 mL∙min^−1^ and a reaction time of 30 min inside the plate flow reactor. The reaction was conducted at 160 °C. The product, a transparent, faintly yellow solution, was collected at the reactor outlet, after passing through a stainless steel Swagelok^®^ R3A series adjustable high pressure valve. This valve was used as a back pressure regulator, in order to set the pressure inside the reactor to between 8 and 10 bar (116 to 145 psi) during operation. From the resulting product solution, the reaction conversion was determined by ^1^H NMR. Afterwards, the solvent was evaporated under reduced pressure to yield a yellow semi-crystalline paste. Detailed reaction conditions and reagent compositions for each experiment in the plate-type flow reactor can be found in Table 3.

## Supporting Information

File 1 Analysis procedures, calculation of *k*-values, reactor performance profiles, reactor fouling, emulsion stabilizing properties, copies of ^1^H and ^13^C NMR and of GC-FID spectra.
