# Supplementary material for: Diels–Alder reactions of myrcene using intensified continuous-flow reactors
Source: Beilstein J Org Chem. 2017 Jan 19;13:120–6. doi: 10.3762/bjoc.13.15 (PMC5301964; doi:10.3762/bjoc.13.15)
Supplement: File 1 — Analysis procedures, calculation of k-values, reactor performance profiles, reactor fouling, emulsion stabilizing properties, copies of 1H and 13C NMR and of GC-FID spectra. [file Beilstein_J_Org_Chem-13-120-s001.pdf]

Supporting Information

for

Diels–Alder reactions of myrcene using intensified continuous-flow reactors

Christian H. Hornung*, Miguel Á. Álvarez-Diéguez, Thomas M. Kohl and John Tsanaktsidis

Address: CSIRO Manufacturing, Bag 10, Clayton South, Victoria 3169, Australia

*Corresponding author

Email: Christian H. Hornung - christian.hornung@csiro.au

Analysis procedures, calculation of k-values, reactor performance profiles, reactor fouling, emulsion stabilizing properties, copies of ^{1}H and ^{13}C NMR and of GC-FID spectra

Analysis of reaction product using gas chromatography:

GC-mass spectra were obtained with a Perkin Elmer Clarus 600 GC mass spectrometer using electron impact ionization in the positive ion mode with an ionization energy of 70 eV. The gas chromatography was performed with a Perkin Elmer Elite-5MS GC column (30 m × 0.25 mm ID, 0.25 μm film thickness), with a temperature program of 40 °C for 2 minutes, then heating at 10 °C/min to 280 °C where the temperature was held for 4 minutes with a split ratio of 70, an injector temperature of 250 °C and the transfer line was set to 250 °C. Ultra-high purity helium was used as the carrier gas with a flow rate of 0.7 mL/min.

GC–FID analysis were performed on an Agilent 6850 Series II gas chromatograph with a split/splitless inlet and a detector temperature of 250 °C. Separation was done on a Grace BPX5 capillary column (25 m × 0.32 mm i.d., 0.50 μm film thickness), with a temperature program of 40 °C for 2 minutes, then heating at 10 °C/min to 280 °C where the temperature was held for 4 minutes with a split ratio of 50 and an injector temperature of 200 °C. High purity helium was used as the carrier gas with a flow rate of 2.4 mL/min.

Calculation of *k* values:

Table S1 shows all k values (reaction rate constants) derived from our experimental data in comparison to k values from the literature. The literature reaction systems were looking at different DE + DEP combinations, different solvents and concentrations and at different reaction temperatures than our experiments, so a direct comparison is not possible. However, the absolute values, especially when comparing entry LIT-1 and LIT-4_{endo} are in the right ballpark and the observed positive trends of k for increasing temperature are also as expected. Below are the mathematical equations used for these calculations:

The reaction type is $A + B \rightarrow C$ where A represents the diene, B the dienophile and C the Diels–Alder adduct or product. The initial kinetic equation of this reaction, *r*, is:

$$r = k \cdot [\mathrm{A}] \cdot [\mathrm{B}] \qquad \text{(S1)},$$

where k is the rate kinetic constant and [A] and [B] are the molar concentrations of the starting materials. As we used starting material ratios of ~1 (ranging from 0.9 to 1.1 of diene to dienophile in the majority of experiments), the following simplification to the kinetic equation can be made using the assumption A ≈ B:

$$r = k \cdot [\mathrm{A}]^2 \qquad \frac{\mathrm{d}[\mathrm{A}]}{[\mathrm{A}]^2} = -k \cdot \mathrm{dt} \qquad \text{(S2)}$$

Assuming 2nd order kinetics, the following can be derived after integration:

$$\int_{\mathrm{A_0}}^{\mathrm{A_i}} \frac{\mathrm{d}[\mathrm{A}]}{[\mathrm{A}]^2} = -\int_0^{\mathrm{t}} k \cdot \mathrm{dt}$$

$$\Rightarrow \quad -\frac{1}{[\mathrm{A_i}]} + \frac{1}{[\mathrm{A_0}]} = -k \cdot \mathrm{t} \quad \Rightarrow \quad \frac{1}{[\mathrm{A_i}]} = k \cdot \mathrm{t} + \frac{1}{[\mathrm{A_0}]} \qquad \text{(S3)}$$

When the conversion of starting material, X_A, is used, k can also be expressed as:

$$k = \frac{X_A}{[A_0]\cdot(1-X_A)\cdot t} \qquad (S4)$$

with $X_A = 1 - \frac{[A_i]}{[A_0]}$.

Using equation S3, the kinetic constant can be derived from a set of data points taken over time from an experiment that was conducted at a certain set of conditions. Figure S1 shows how the k value was derived from one set of data (entry 2.1 – see Tables 2 or S1)

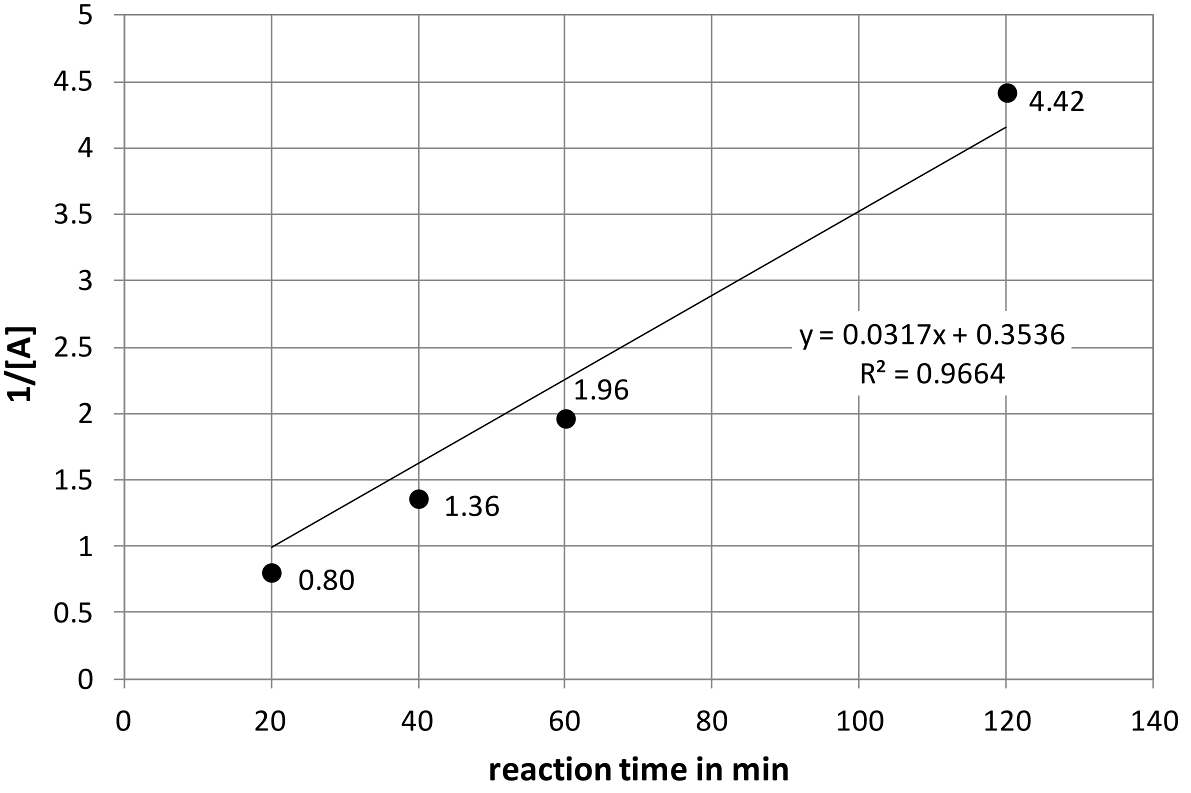

Figure S1. Diagram showing derivation of k values on one example: entry 2.1 (see Table S1).

Table S1: Comparison of the *k* values from these experiments with literature values.

entry	DE[a]	DEP[b]	solvent	$c_{DE,0}$ [mol L^{-1}]	T [°C]	$k \times 10^6$ [L mol^{-1} s^{-1}]	reference
2.1	myrcene	AA	EtOAc	2.8	120	528	-
2.2	myrcene	AA	EtOAc	2.8	140	3440	-
2.3	myrcene	AA	toluene	2.8	100	267	-
2.4	myrcene	AA	toluene	2.8	120	1140	-
2.5	myrcene	AA	toluene	2.8	140	4750	-
2.6	myrcene	AA	toluene	2.9	160	27050	-
LIT-1	CP	DMF	EtOAc	0.37	175	967	[S1]
LIT-2_{endo}	furan	MA	MeCN	*neat*	25	17.5	[S2]
LIT-2_{exo}	furan	MA	MeCN	*neat*	25	31.0	[S2]
LIT-3_{endo}	furan	MI	MeCN	*neat*	25	0.82	[S2]
LIT-3_{exo}	furan	MI	MeCN	*neat*	25	0.55	[S2]
LIT-4_{endo}	furan	MI	MeCN	*neat*	64	167	[S2]
LIT-4_{exo}	furan	MI	MeCN	*neat*	64	1.37	[S2]

a) DE = diene, CP = cyclopentadiene; b) DEP = dienophile, AA = acrylic acid (see Table 1, **2b**), DMF = dimethyl fumarate, MA = maleic anhydride (see Table 1, **2a**), MI = maleimide.

Reactor performance of the Chemtrix Plantrix® MR260 flow reactor:

As described in the experimental section of the manuscript, the Chemtrix Plantrix® MR260 flow reactor was operated in conjunction with two Teledyne Isco D-series dual syringe pumps, an SSI Prep 100 dual piston pump and a Lauda Integral XT 150 heater/chiller unit. The experimental set-up is shown in Figure S2 and a close up of the 3M™ silicon carbide reactor module [S3] with outer cover (without outer cover is shown in Figure S3).

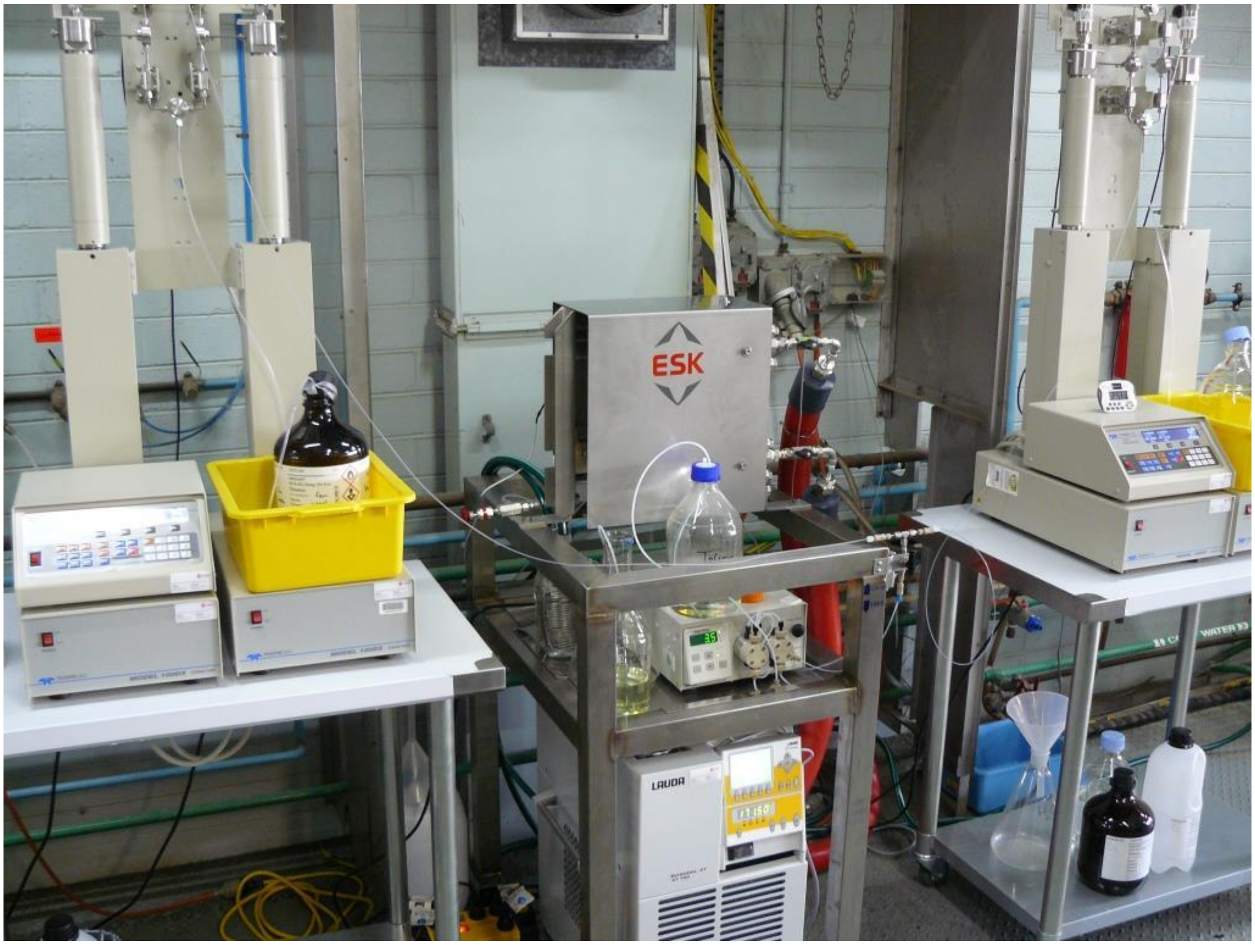

Figure S2: Experimental set-up for the Diels–Alder reaction of myrcene and acrylic acid in a Chemtrix Plantrix® MR260 flow reactor.

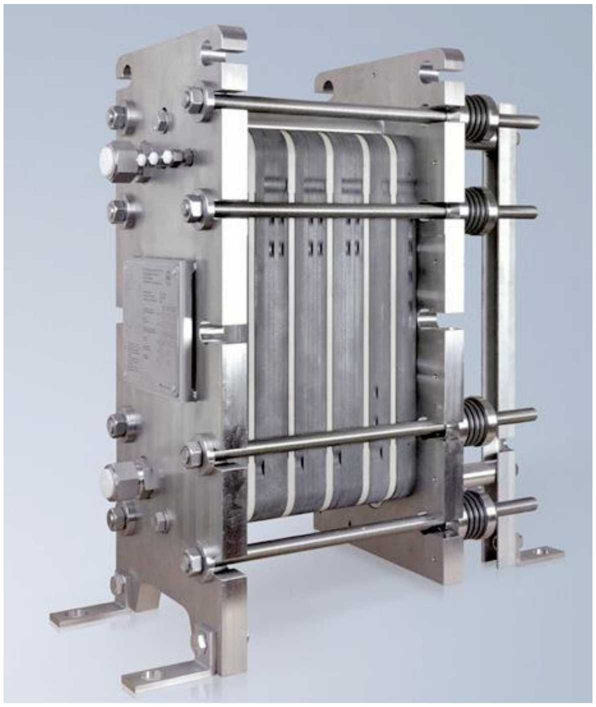

Figure S3: 3M^{TM} silicon carbide reactor module - Chemtrix Plantrix® MR260 continuous-flow reactor.[15]

In order to quantify the consistency of the product solution exiting the Chemtrix Plantrix® MR260 continuous-flow reactor, a set of ^{1}H NMR samples were taken at the reactor outlet, over the entire duration of an experiment. From this a conversion profile was established. At the beginning the conversion rises steeply as the toluene is replaced by reaction mixture before stabilizing around 93% at steady state conditions, and then drops off again after 160 min as the reactants are replaced by toluene. The profile is very uniform and the front and tail are steep, suggesting that the residence time distribution inside the reactor is narrow and close to plug flow (see Figure S4).

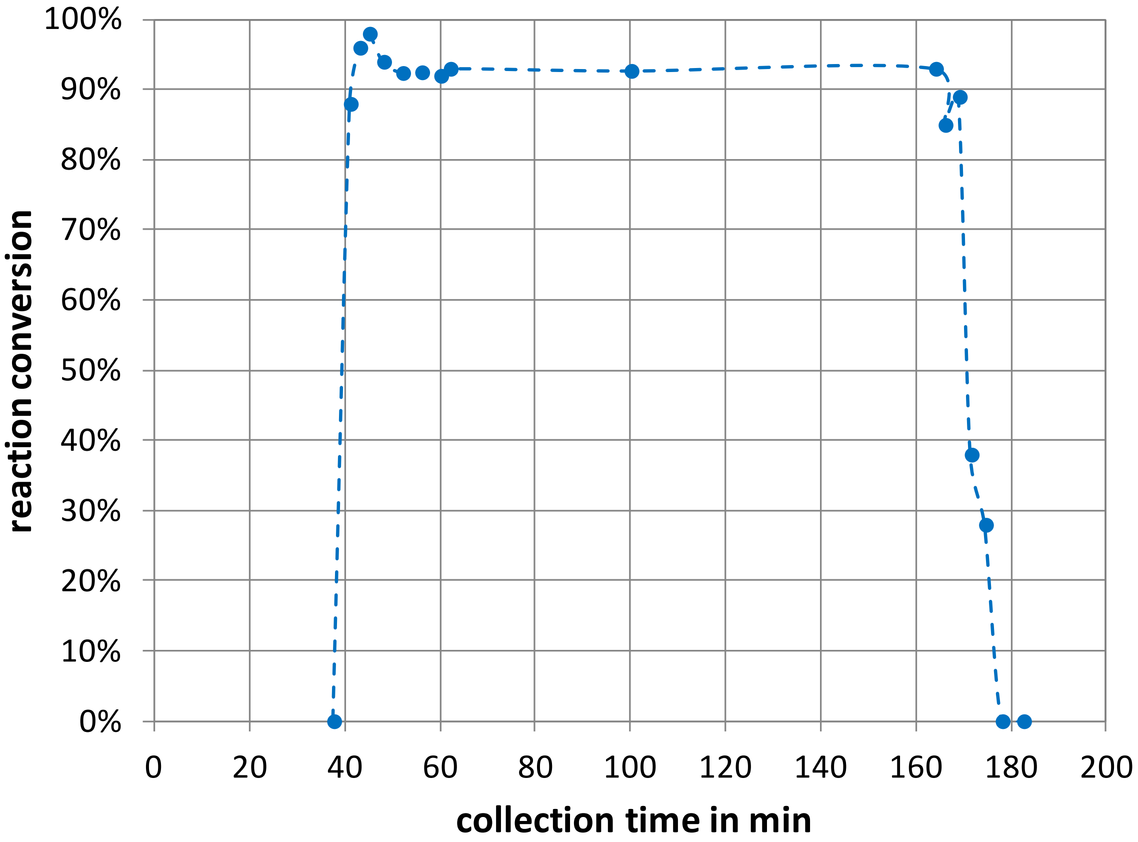

Figure S4: Conversion profile for the Diels–Alder reaction of myrcene and acrylic acid in toluene at 130 °C inside the Chemtrix Plantrix® MR260 flow reactor (entries 3.7, Table 3).

Reactor fouling due to co-polymerization of acrylic acid and myrcene:

During our scale-up investigations of the continuous process, from the laboratory scale Vapourtec R2/R4 flow reactor system to the pilot scale Chemtrix Plantrix® MR260 flow reactor, we also investigated another reactor design, namely a Salamander tubular flow reactor from Cambridge Reactor Design (reactor volume: 108 mL). This reactor consisted of 6 mm ID stainless steel tubes, fitted with stainless steel static mixers, and was operated with a set of HPLC pumps. The heating of this reactor is electrical and further details can be found in a previous publication, where we used it for the synthesis of poly acrylic acid amongst others [S4]. When trying to perform the Diels–Alder reaction of myrcene and acrylic acid in this reactor, we observed first a pressure increase at several minutes after start of the reaction, which was caused by fouling occurring in the entrance section of the reactor, and which ultimately led to complete blockage of the tube at this point. The blockage occurred over a length of ~10 cm after the inlet (see Figure S5). After replacing the blocked tube with a new one, the reaction was repeated and similar complications occurred: pressure build-up due to fouling leading to complete blockage of the reactor. We tried to investigate the cause of this blockage and tried to analyze the unwanted side-product. The side-product was a white powder which was compacted to a hard solid inside the tube and it showed no solubility in any of the common solvents we tried (water, alcohols, acetone, EtOAc, toluene, dimethyl sulfoxide). Hence the only simple analysis we could perform was FTIR of the white powder. The resulting spectrum showed large similarities to library spectra of polyacrylic acid. We assume that upon heating a polymer side product was formed inside the tubular steel reactor, which mainly consisted of acrylic acid. We further assume that this polyacrylic acid was cross linked with myrcene, which would explain the low solubility of the material. As this side product was not observed in the Vapourtec R2/R4 flow reactor or the

Chemtrix Plantrix® MR260 flow reactor, we followed that the reactor material must have catalyzed this side reaction. While the reactor modules of the Vapourtec R2/R4 are made from PFA tubing and the reactor module of the Chemtrix Plantrix® MR260 is made from 3M™ silicon carbide, the Salamander reactor was made from 316 stainless steel. To this date, this seems to be the most plausible explanation, given that we do not have a detailed understanding of the processes which lead to the reactor fouling. No further Diels–Alder reactions on the stainless steel reactor were conducted after this incident.

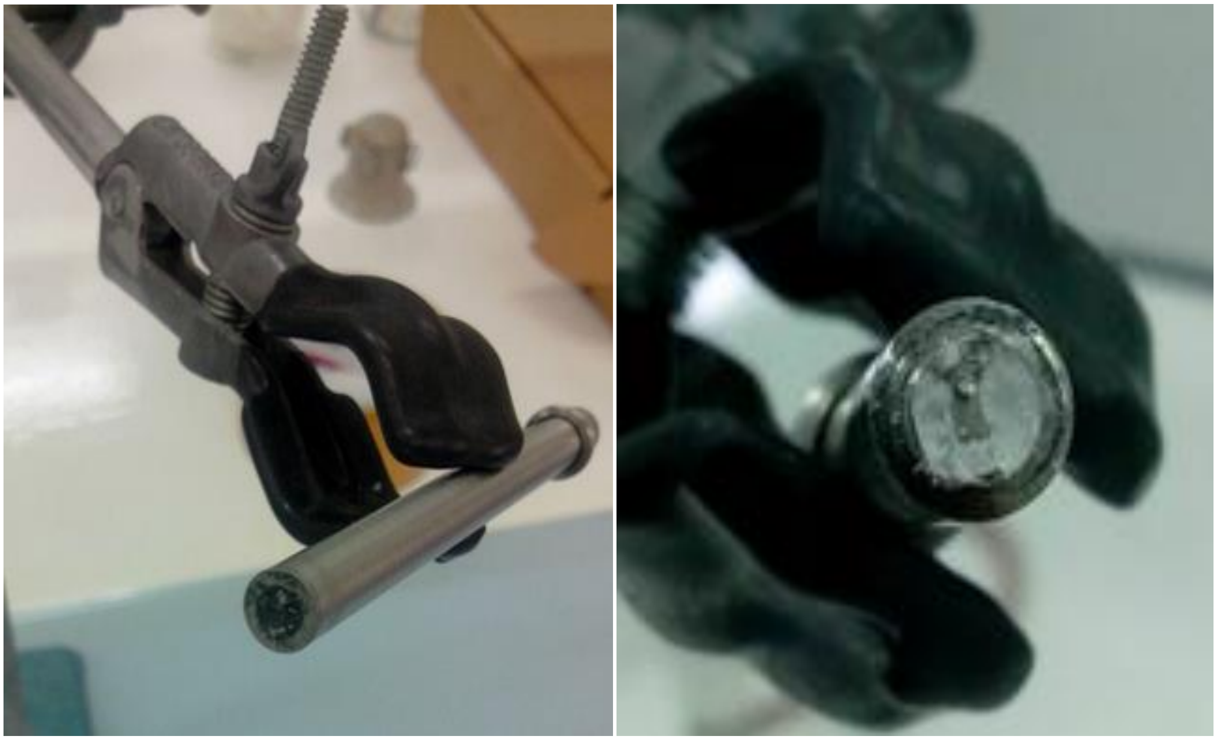

Figure S5: Images of a blocked 6 mm i.d. reactor tube of a tubular stainless steel reactor (with static mixer inserts); the white material is poly(acrylic acid-co-myrcene).

Emulsion stabilizing properties of Diels–Alder adduct:

The surfactant properties of the Diels–Alder adduct of myrcene and acrylic acid were investigated using set of experiments with which the ability of this compound to stabilize an emulsion was studied. The purified yellow semi-crystalline product from experiment 3.5 (see Table 3) was used for this set of experiments. Twelve samples containing a 1:1 mixture of water and a non-water miscible organic solvent (toluene, EtOAc or DCM) and small amounts of starting material and Diels–Alder adduct were prepared according to Table S2. The samples were shaken by hand for several seconds until an emulsion was formed. This emulsion was then left to settle and form two separate layers again, during which time was measured.

Table S2: Compositions of 1:1 v/v mixtures of water and an organic solvent (toluene, EtOAc or DCM) + small quantities of starting materials or Diels–Alder adduct.

**Sample**	**Separation time**
**T1** - water-toluene mixture + 100 mg of myrcene	60 s
**T2** - water-toluene mixture + 100 mg of acrylic acid	20 s
**T3** - water-toluene mixture + 100 mg of Diels–Alder adduct	23 min to 1.25 h
**T4** - water-toluene mixture	20 s
**E1** - water-EtOAc mixture + 100 mg of myrcene	100 s
**E2** - water-EtOAc mixture + 100 mg of acrylic acid	70 s
**E3** - water-EtOAc mixture + 100 mg of Diels–Alder adduct	24 min to 2.5 h
**E4** - water-EtOAc mixture	70 s
**D1** - water-DCM mixture + 100 mg of myrcene	20 s
**D2** - water-DCM mixture + 100 mg of acrylic acid	30 s
**D3** - water-DCM mixture + 100 mg of Diels–Alder adduct	14 min to 2.25 h
**D4** - water-DCM mixture	10 s

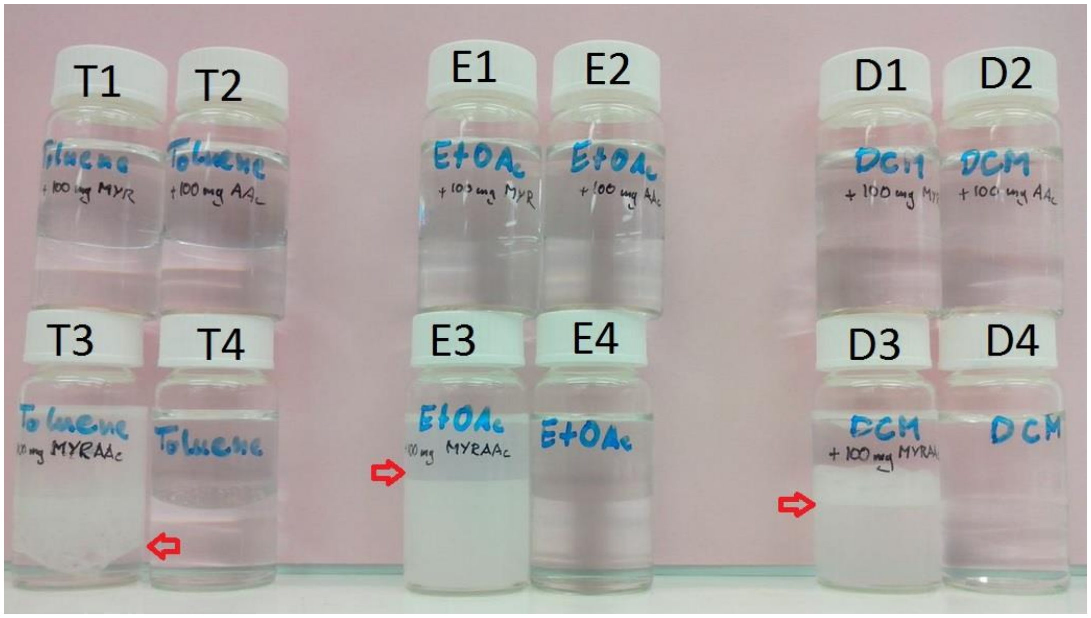

Figure S6: Image of a series of mixtures of water and an organic solvent, some containing starting material and others Diels–Alder adduct. The image was taken 18 min after agitation, the red arrows indicate the forming phase boundary between two emulsion layers for the samples containing Diels–Alder adduct

The picture in Figure S6 was taken 18 min after agitation. At this time all emulsions have separated but the ones containing the Diels–Alder adduct. The samples containing acrylic acid (T2, E2, D2) and the control samples without any starting material or product (T4, E4, D4) completely separated within 10 to 70 s and the samples containing myrcene (T1, E1, D1) separated within 20 to 100 s. In contrast, for the samples containing the Diels–Alder adduct (T3, E3, D3) it took between 1.25 and 2.5 h before the majority of the sample was separated; at this point there were still small pockets of emulsion left. The onset of separation was between 14 and 24 min; with all other samples the onset was instantaneous. The red arrows indicate the forming interfacial boundary between two emulsion layers for the samples containing Diels–Alder adduct. This large difference in emulsion stabilizing time between a few seconds without and

several hours with the Diels–Alder adduct is a clear indication of the good surfactant properties of the product. Further tests need to be carried out in order to quantify the surfactant properties of these materials in more depth.

Copies of ^{1}H and ^{13}C NMR spectra

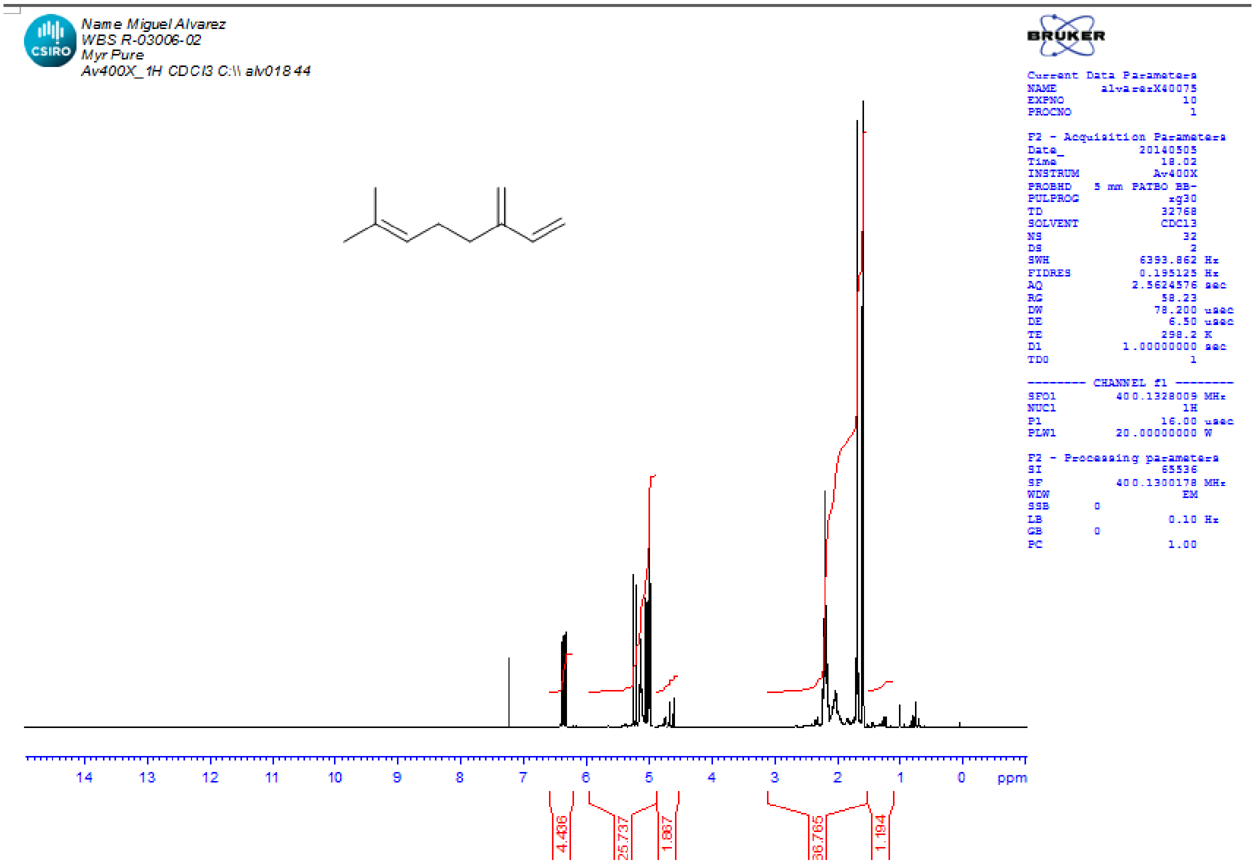

Figure S7: ^{1}H spectrum of myrcene (**1**).

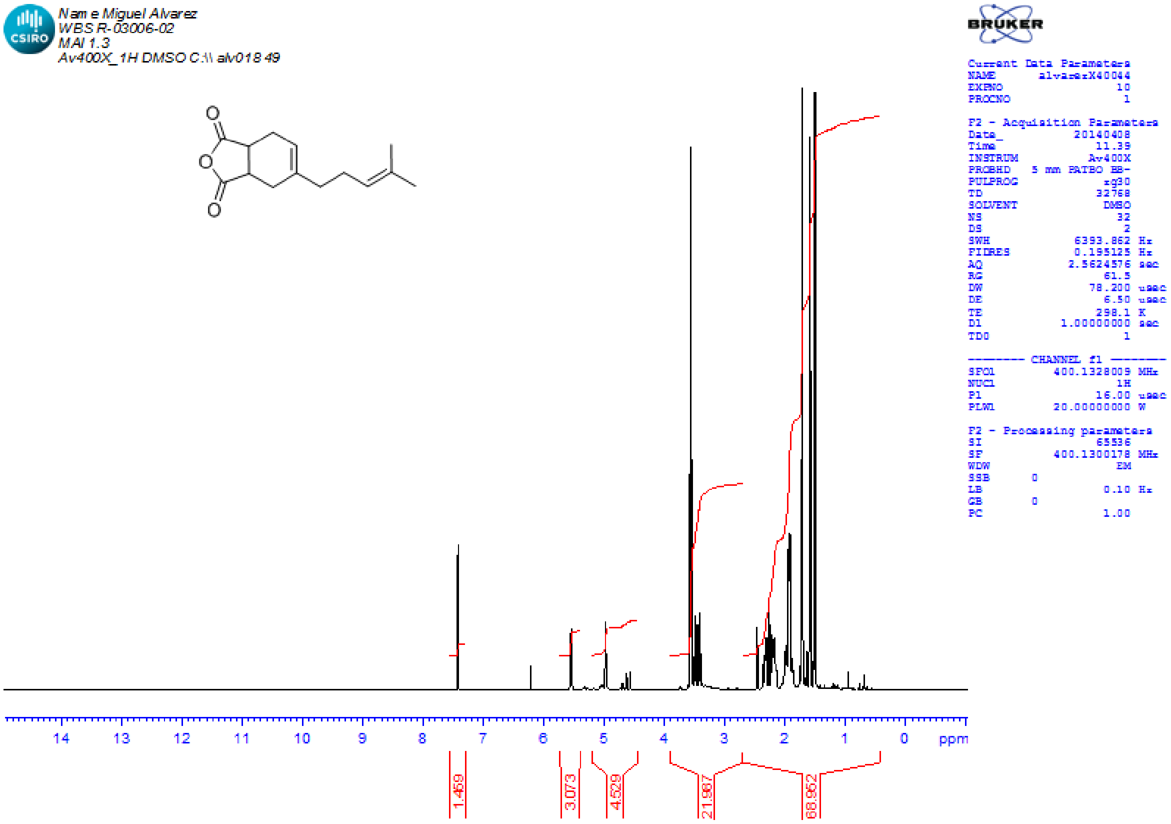

Figure S8: ^{1}H spectrum of Diels-Alder adduct of myrcene and maleic anhydride, **2a**.

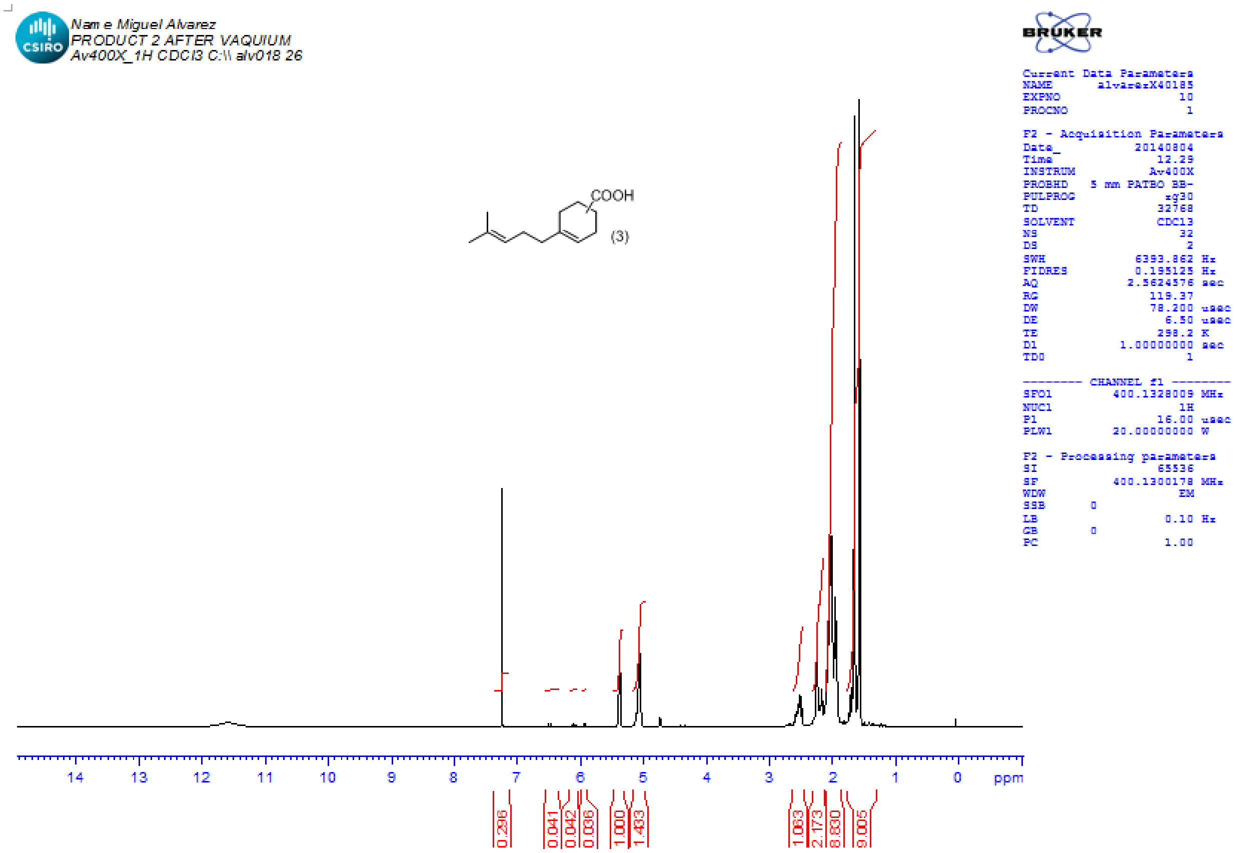

Figure S9: ^{1}H spectrum of Diels-Alder adduct of myrcene and acrylic acid, **2b**.

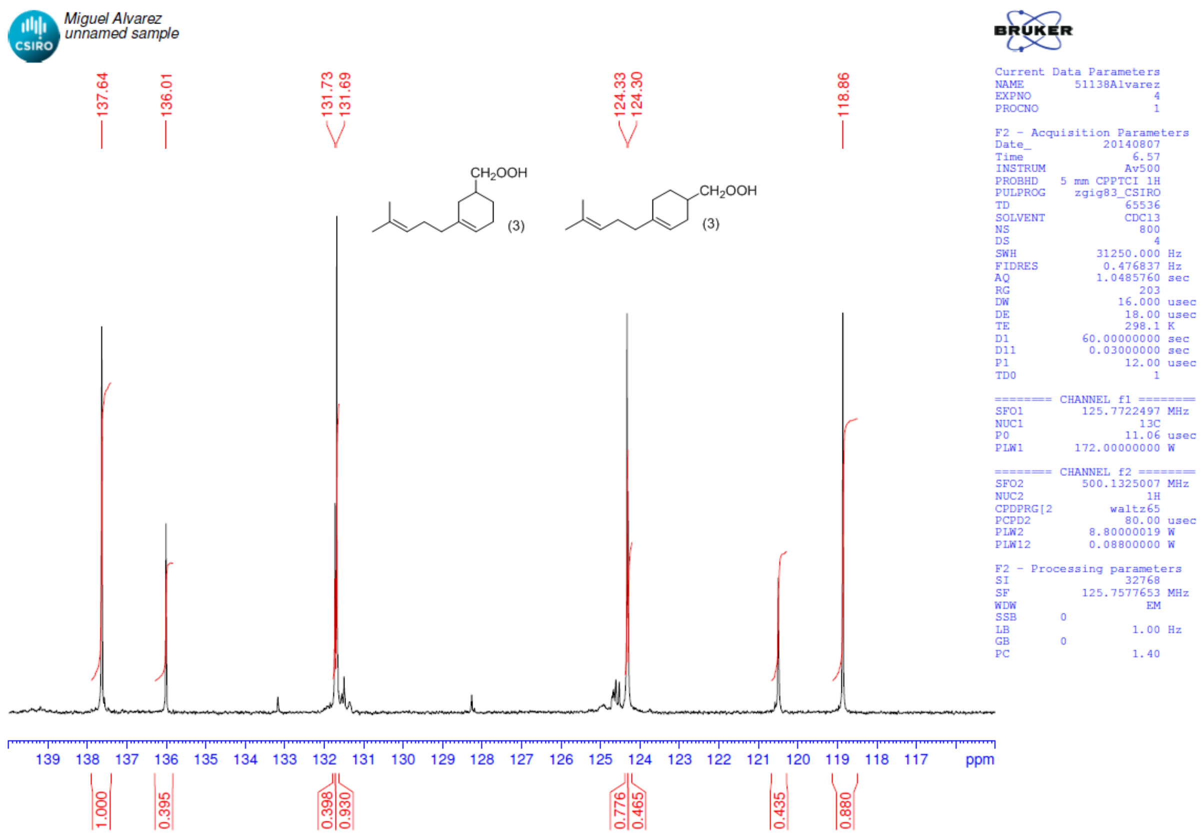

Figure S10: ^{13}C spectrum of Diels-Alder adduct of myrcene and acrylic acid, **2b**.

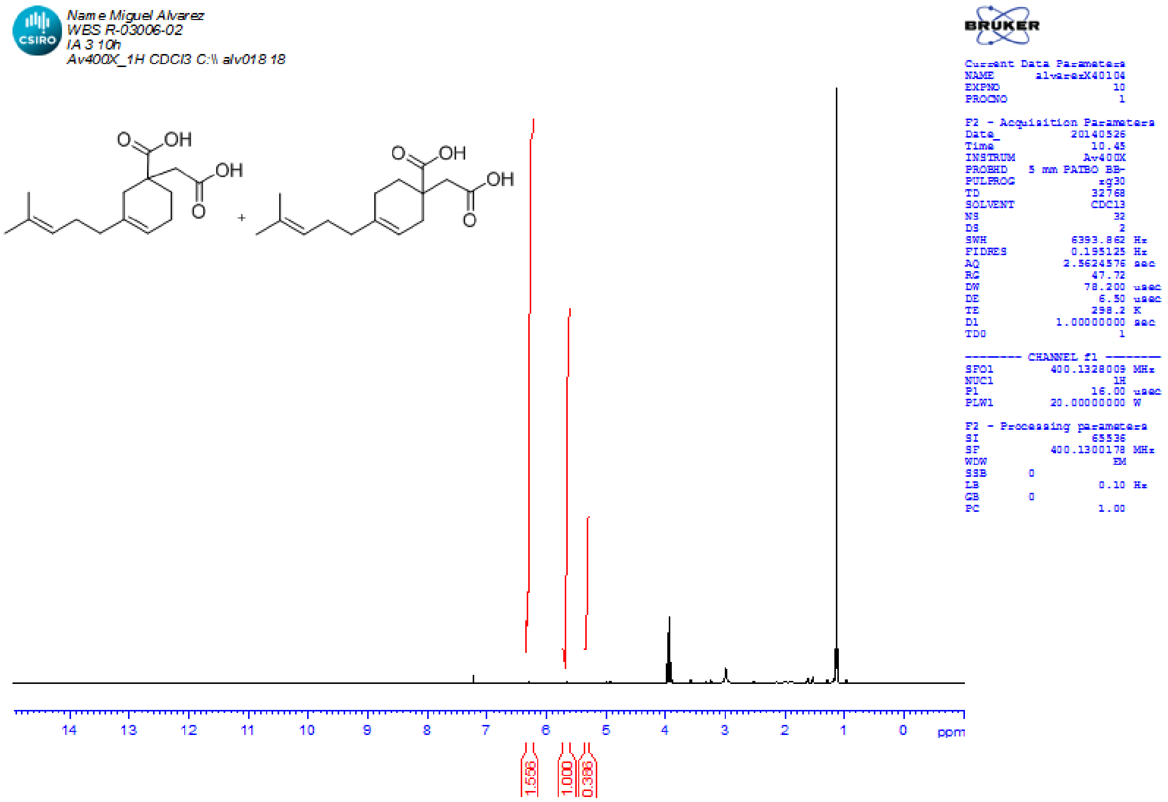

Figure S11: ^{1}H spectrum of Diels-Alder adduct of myrcene and itaconic acid, **2c**.

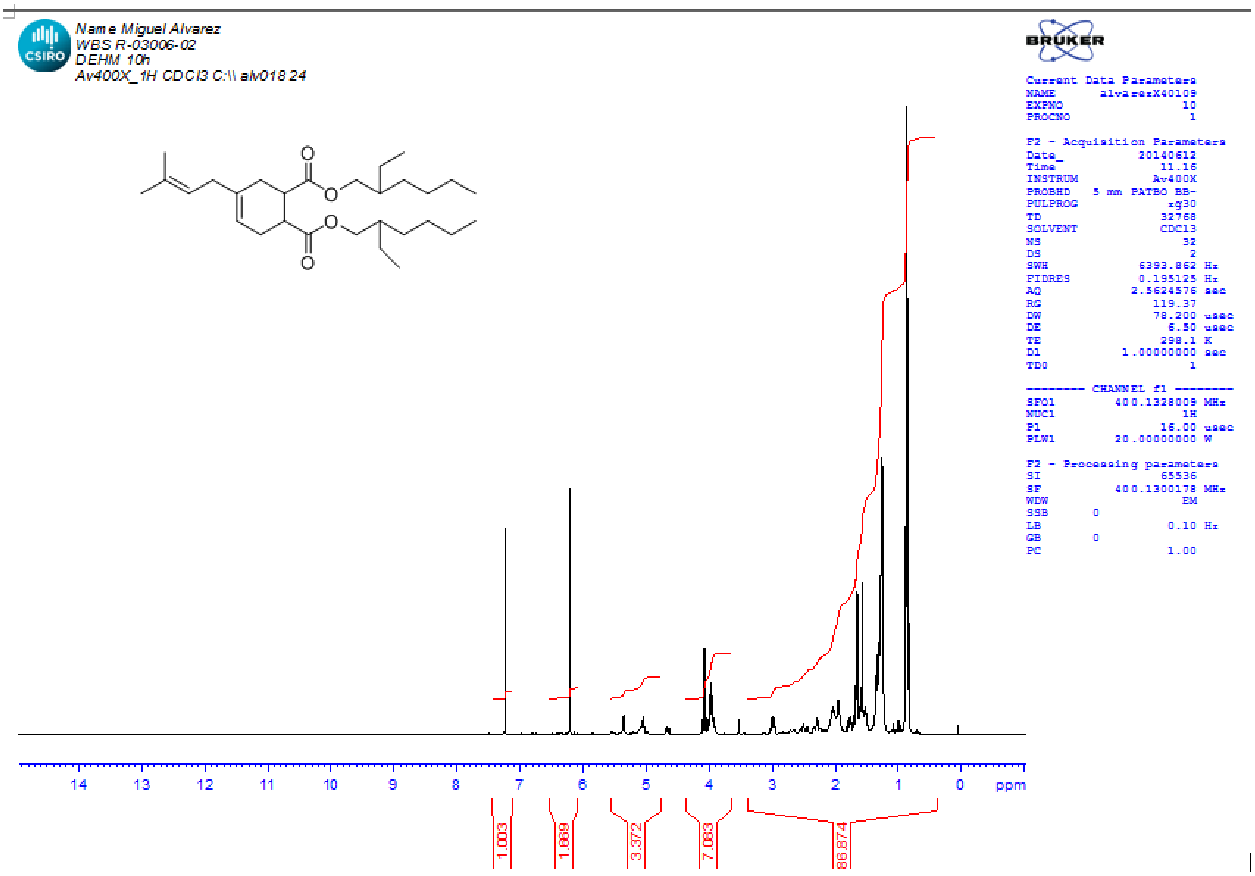

Figure S12: ^{1}H spectrum of Diels-Alder adduct of myrcene and bis(2-ethylhexyl) maleate, **2e**.

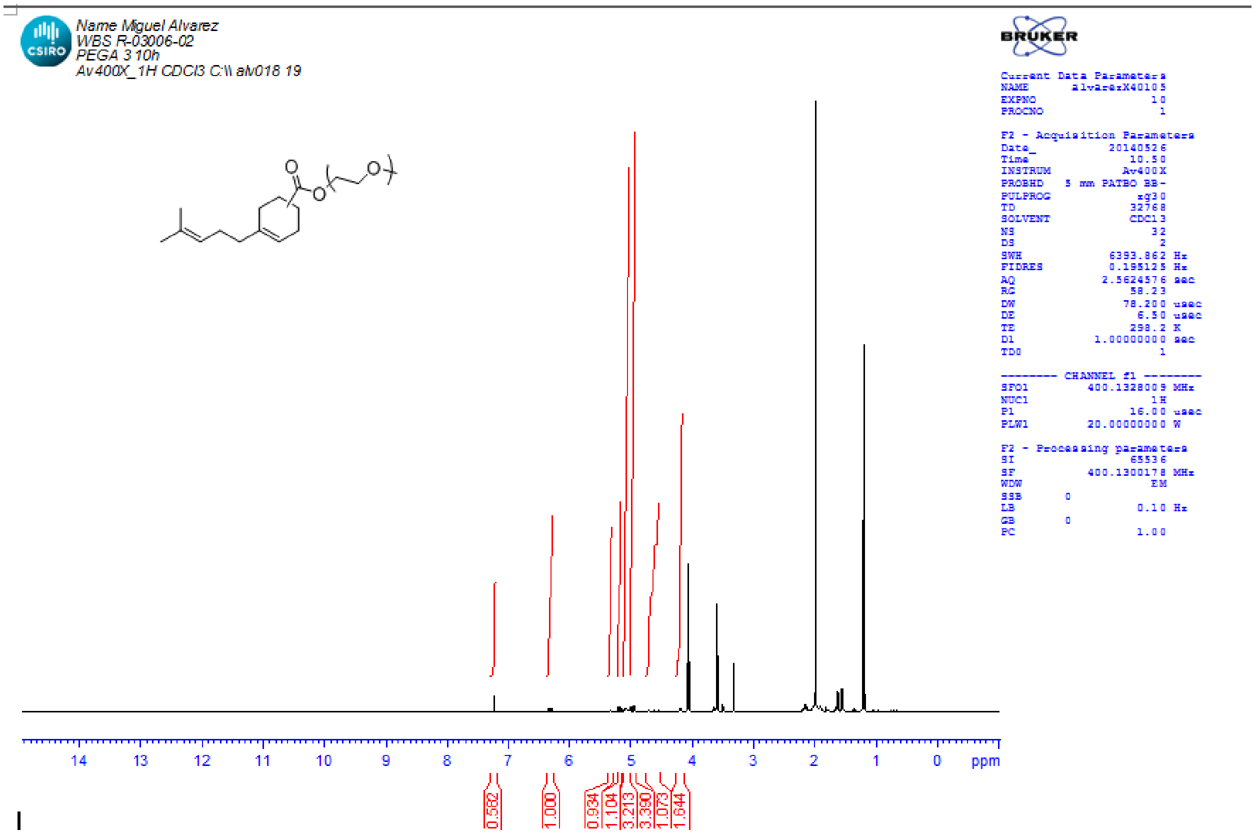

Figure S13: ^{1}H spectrum of Diels-Alder adduct of myrcene and poly(ethylene glycol) methyl ether acrylate, **2g**.

Copies of GC-FID spectra

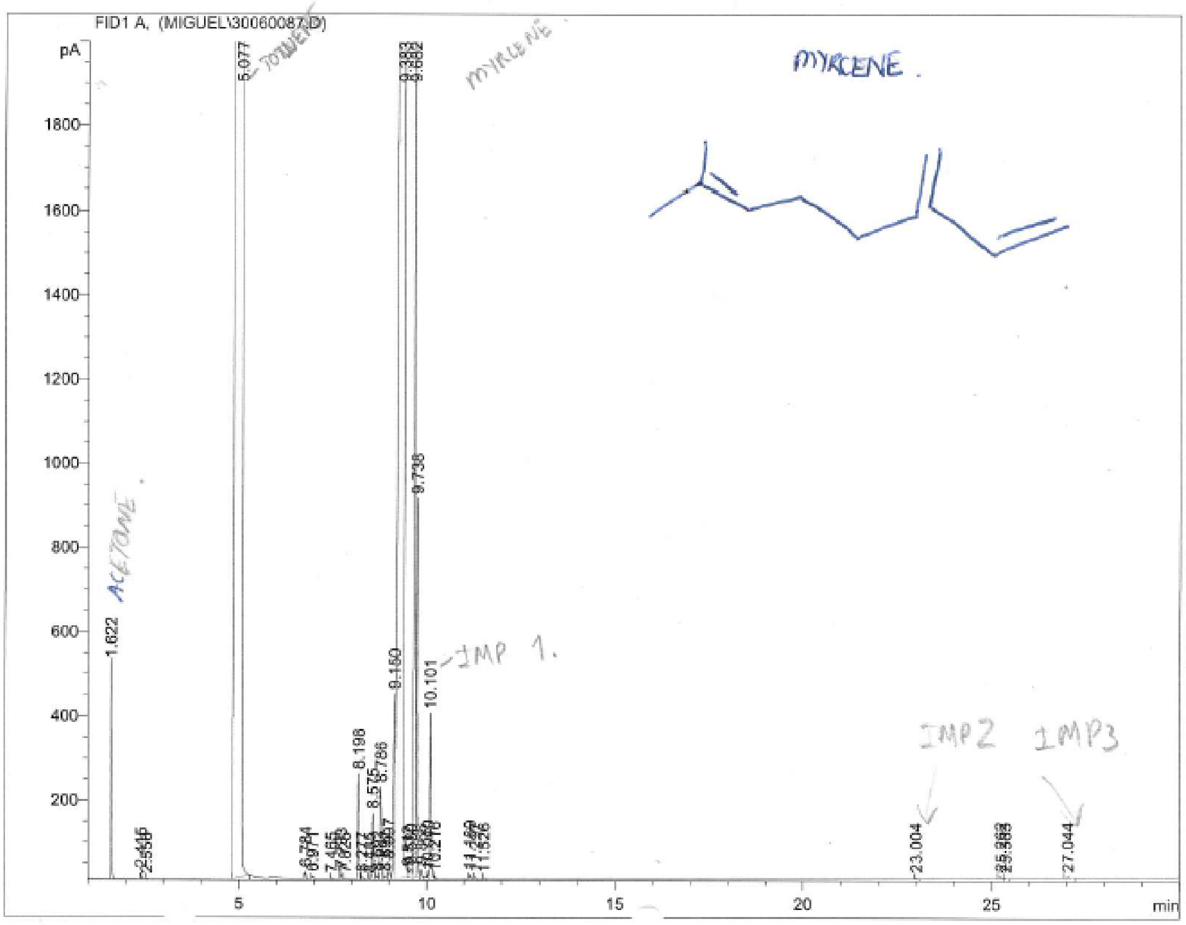

Figure S14: GC-FID spectrum of myrcene.

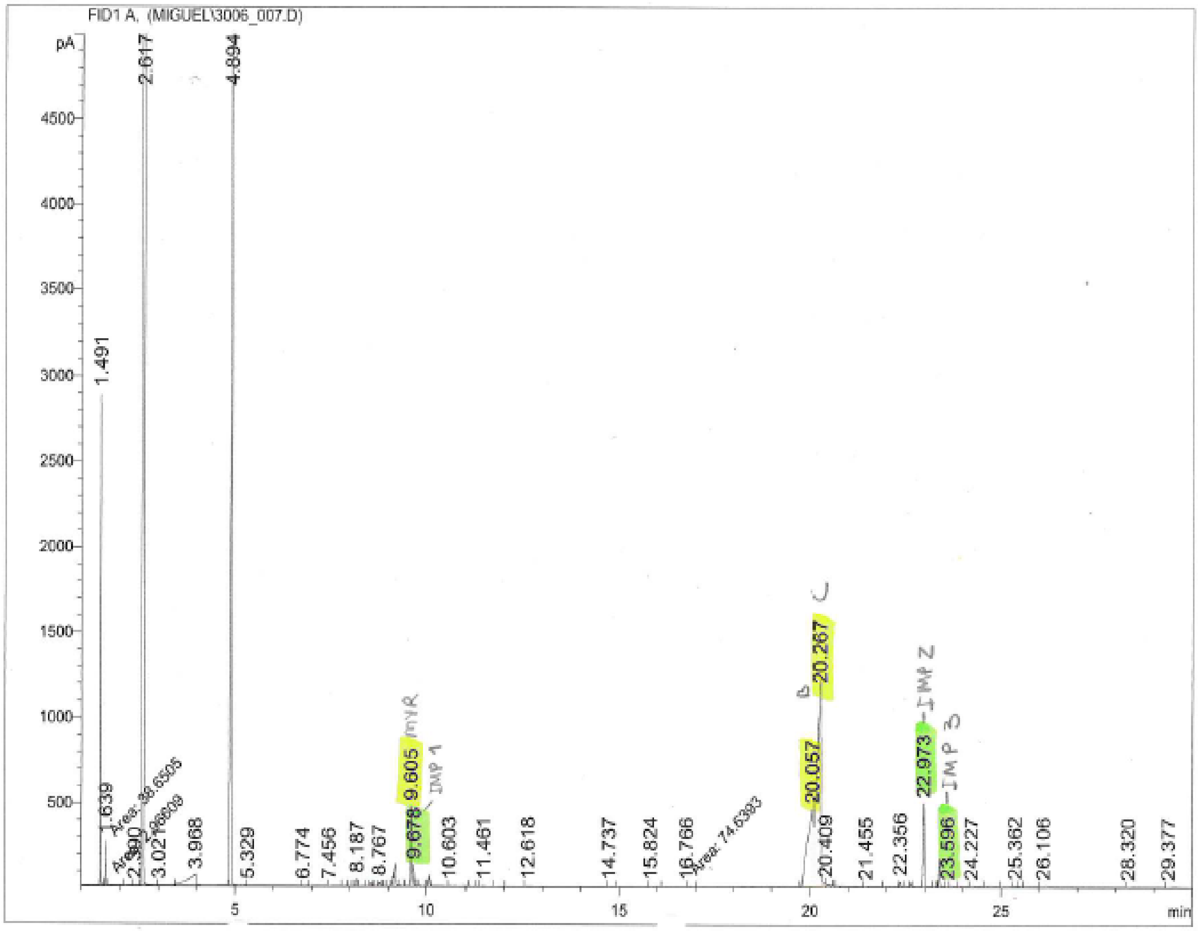

Figure S15: GC-FID spectrum of Diels-Alder adduct of myrcene and acrylic acid, **2b**.

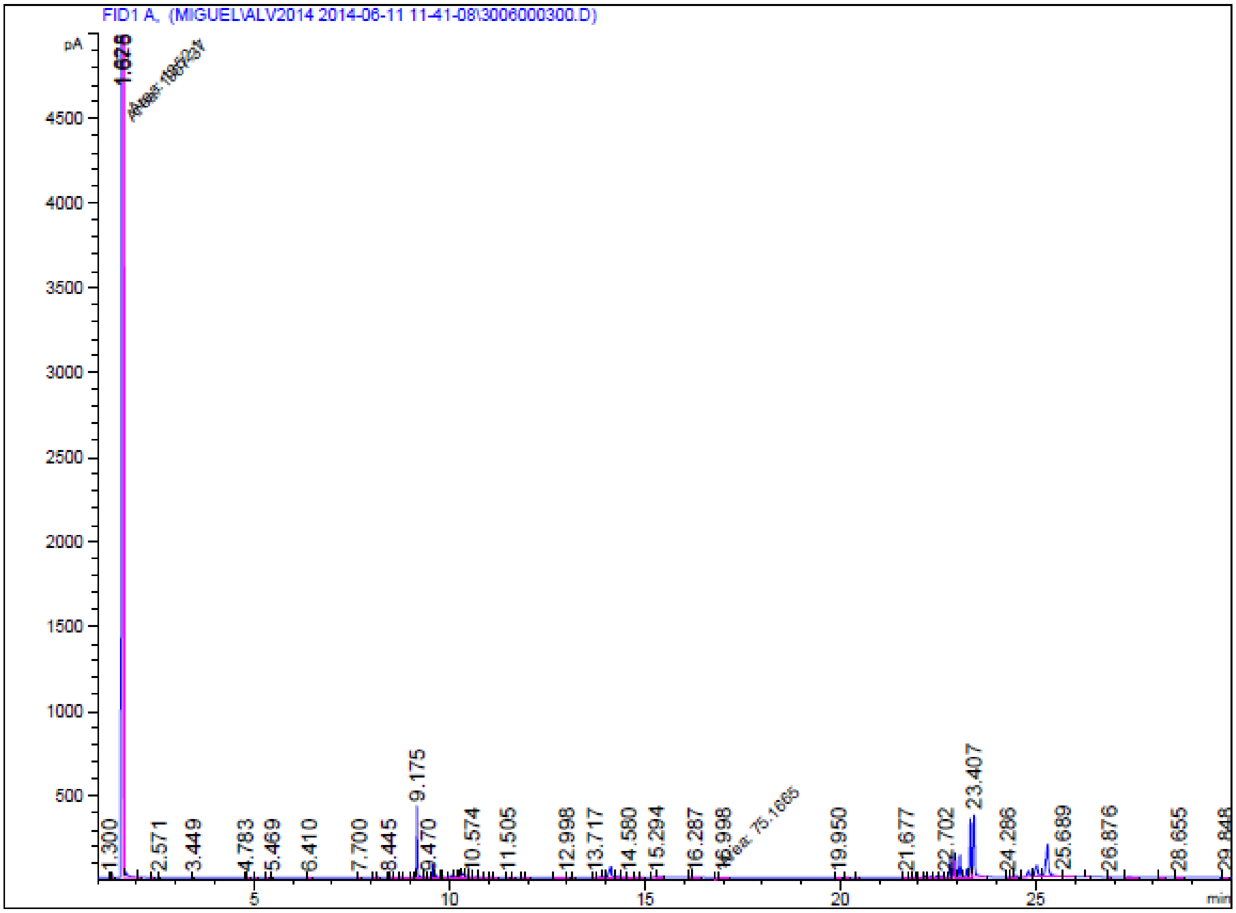

Figure S16: GC-FID spectrum of Diels-Alder adduct of myrcene and itaconic acid, **2c**.

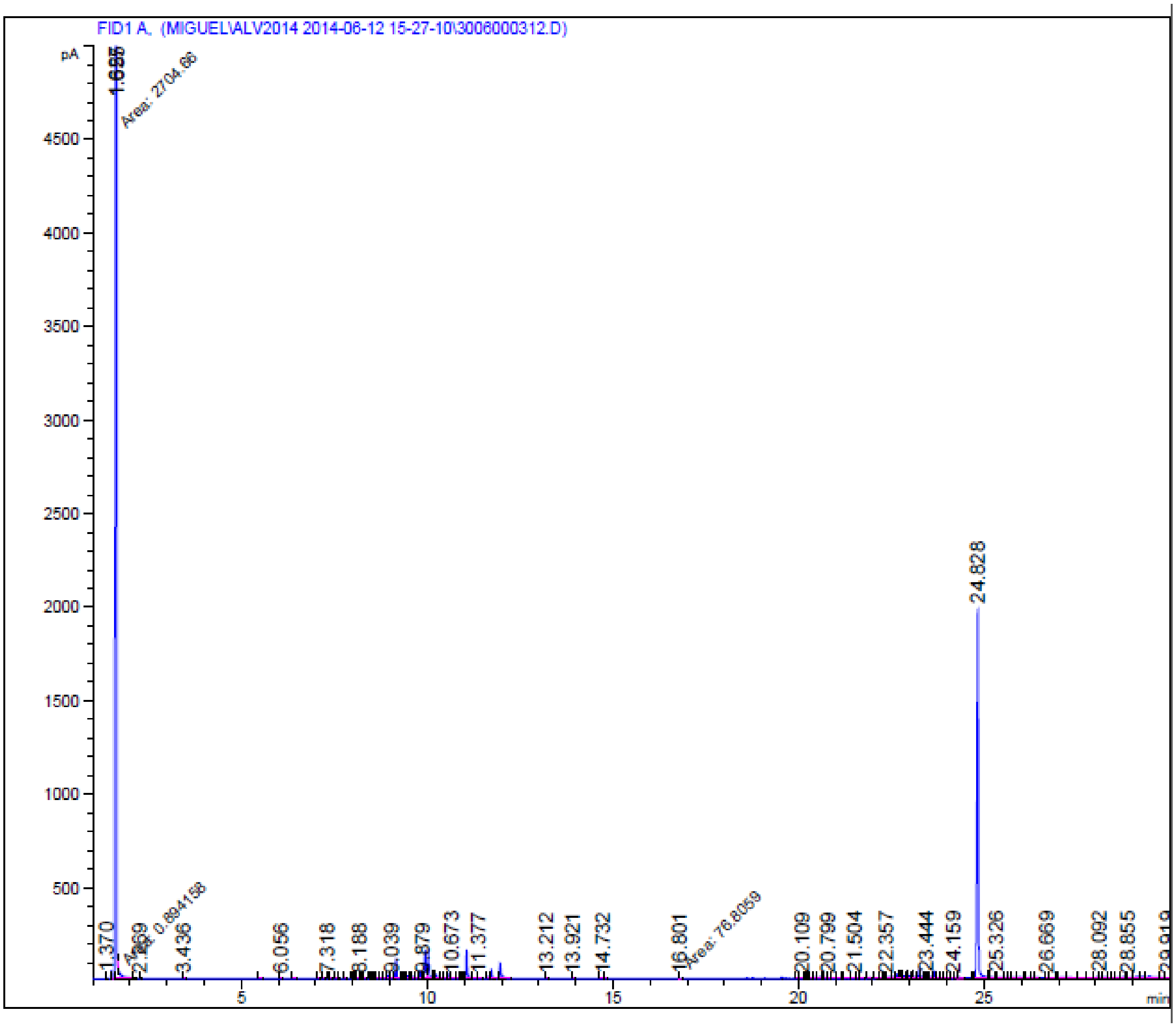

Figure S17: GC-FID spectrum of Diels-Alder adduct of bis(2-ethylhexyl) maleate, **2e**.

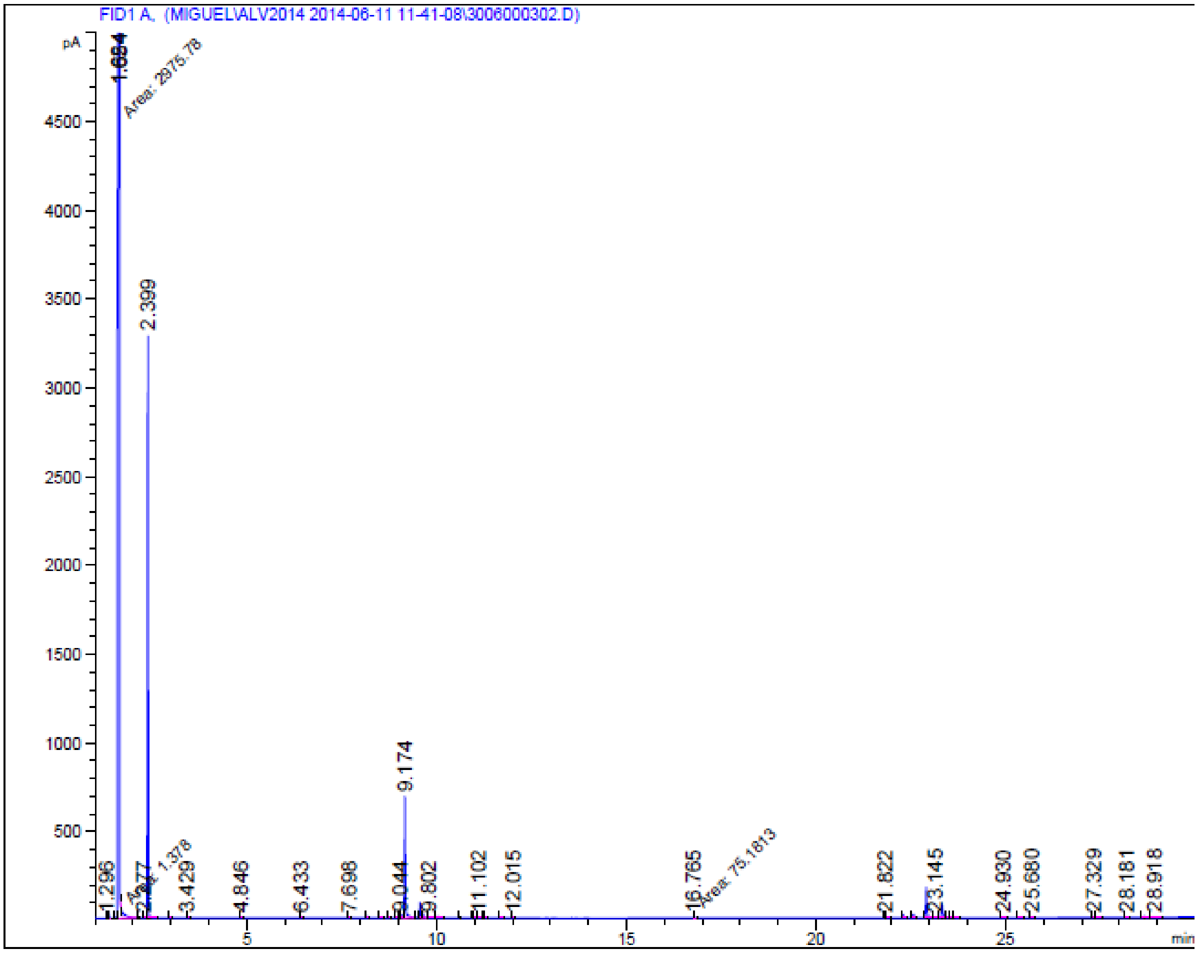

Figure S18: GC-FID spectrum of Diels-Alder adduct of myrcene and poly(ethylene glycol) methyl ether acrylate, **2g**.

References

S1. Silvestri, M. G.; Dills, C. E. *J. Chem. Educ.* **1989**, *66*, 690.

S2. Rulíšek, L.; Šebek, P.; Havlas, Z.; Hrabal, R.; Čapek, P.; Svatoš, A. *J. Org. Chem.* **2005**, *70*, 6295.

S3. Vapourtec Ltd., 2016, http://www.vapourtec.co.uk/home

S4. Ehrfeld, W.; Hessel, V.; Löwe, H. *Microreactors: New Technology for Modern Chemistry.* Wiley-VCH Verlag GmbH: Weinheim, 2000.
